# Improved Ablation Efficiency in PVI Guided by Contact Force and Local Impedance: Chronic Canine Model

**DOI:** 10.3389/fphys.2021.808541

**Published:** 2022-01-10

**Authors:** Sarah R. Gutbrod, Allan Shuros, Vijay Koya, Michelle Alexander-Curtis, Lauren Lehn, Kimberly Miklos, John Paul Mounsey, Jason D. Meyers

**Affiliations:** ^1^Boston Scientific Corp., Marlborough, MA, United States; ^2^Department of Internal Medicine and Cardiovascular Medicine, University of Arkansas for Medical Sciences, Little Rock, AR, United States; ^3^Department of Clinical Cardiac Electrophysiology, Iowa Heart Center, West Des Moines, IA, United States

**Keywords:** catheter ablation, high power short duration, impedance, atrial fibrillation, radio frequency

## Abstract

**Background:** The purpose of this study was to assess the effect local impedance (LI) has on an ablation workflow when combined with a contact force (CF) ablation catheter.

**Methods:** Left pulmonary vein isolation was performed in an *in vivo* canine model (*N* = 8) using a nominal (30 W) or an elevated (50 W) power strategy with a CF catheter. The catheter was enabled to measure LI prior to and during ablation. LI was visible for only one of the vein isolations.

**Results:** Chronic block was achieved in all animals when assessed 30 ± 5 days post-ablation procedure with a median LI drop during RF ranging from 23.0 to 34.0 Ω. In both power cohorts, the median radiofrequency (RF) duration decreased if LI was visible to the operator (30 W only CF: 17.0 s; 30 W CF + LI: 14.0 s, *p* = 0.009; 50 W only CF: 6.0 s; 50 W CF + LI: 4.0 s, *p* = 0.019). An inverse relationship between the LI prior to RF delivery and the RF duration required to achieve an effective lesion was observed. There was no correlation between the magnitude of the applied force and the drop in LI, once at least 5 g was achieved.

**Conclusions:** An elevated power strategy with the context of CF and LI led to the most efficient titration of successful RF energy delivery. The combination of feedback allows for customization of the ablation strategy based on local tissue variation rather than a uniform approach that could potentially lead to overtreatment. Higher LI drops were more readily achievable when an elevated power strategy was utilized, especially in conditions where the catheter was coupled against tissue with low resistivity. Clinical study is warranted to determine if there is an additive safety benefit to visualizing the dynamics of the tissue response to RF energy with LI when an elevated power strategy is used.

## Introduction

Radiofrequency (RF) ablation has been performed for decades for the treatment of cardiac arrhythmias. Since its introduction there has been a continuous evolution of workflow strategies and additional feedback mechanisms aimed to aid in the optimal titration of energy delivery. The heat that creates the desired lesions is proportional to the energy driven through the tissue and the inherent biophysical parameters of the target tissue, where tissue with increased resistivity generates more heat as the RF current passes through it. The former is predominantly under the control of the operator while variation in the latter must be overcome to achieve consistent lesion formation. Due to the nature of the design of RF ablation systems, the current that reaches the tissue is dependent on both the generator output and the coupling between the electrode and the target tissue or surrounding blood pool (Haines, 1991). Additionally, the resulting temperature reached within the tissue for a given amount of current is altered by the resistive load of that tissue locally.

In the current clinical state of the art, power, time, and tissue contact are monitored to balance the formation of effective transmural lesions with the risk of adverse effects (i.e., cardiac perforation, steam pops, collateral thermal damage, and thrombus formation). With the introduction of force sensing catheters, physicians gained access to additional feedback on the mechanical interaction between the catheter tip and tissue to improve consistency of tissue coupling. Clinical studies have demonstrated improvements in procedural time, reduced fluoroscopy time, higher rate of generator impedance drops, and reduced RF application time for acute isolation of the pulmonary veins (PV) with the use of force-sensing catheters compared to non-force-sensing catheters (Martinek et al., 2012; Neuzil et al., 2013; Kimura et al., 2014; Marijon et al., 2014; Natale et al., 2014; Sigmund et al., 2015). Yet despite numerous trials and many years of clinical experience, establishing a direct connection between the use of Contact Force (CF) feedback and long-term lesion durability has been difficult. This is likely because force alone does not reflect the variation in the resistive load of the tissue or the effect of local blood flow dynamics that alter heating.

In more recent years, higher power settings have been explored in a further effort to improve consistency of ablation. Although initial studies on an elevated power for a shorter duration strategy have focused on improved efficiency, minimizing the confounding effect of catheter instability and overcoming the challenges of encroaching edema (Barkagan et al., 2018), the increase in current may also have the added benefit of creating more predictable heating in other physiological conditions of decreased resistivity. In order to effectively harness this potential, feedback on the resistive load and how the tissue is dynamically responding to the input RF energy is critical to titrate RF duration to effect.

Previously, a catheter was introduced that combines CF and a measure of LI (Garrott et al., 2020). The LI metric builds on the principles of monopolar generator impedance measurements, enhancing the dynamic range and the correlation with respect to lesions *in vivo* by providing dynamic feedback on volumetric heating. The method for acquiring LI has been described in detail on two catheter platforms (Sulkin et al., 2018; Garrott et al., 2020). The value of measuring a LI has been evaluated clinically on a catheter without contact force retrospectively (Gunawardene et al., 2019) and prospectively (Das et al., 2021). Here, three electrodes from the catheter are used to create a local electric field and measure the potential, where the tip electrode is included in both the driving and sensing circuit. By removing the influence of any electrodes at a distance, the measurement is dependent on only the local dielectric changes. With this investigation we compare the use of 30 W to an elevated power strategy at 50 W within the context of CF alone or the combination of CF and Local Impedance (LI) during isolation of the left PVs in a canine model. By comparing these cohorts, we can assess the additive effect of LI on the efficiency of ablation strategies. Additionally, the animals were survived for 30 ± 5 days to confirm that any acute efficiency advantages still led to mature lesion durability.

## Methods

Eight healthy mongrel canines (4 M/4 F, 9–13 months old, weighing ≥25 kg) were used for this study (Oak Hill Genetics). Animal use for this investigation was approved by the Institutional Animal Care and Use board of Boston Scientific Corp. To minimize bias the study was conducted in compliance with 21 CFR Part 58 Good Laboratory Practice guidelines with an independent auditor.

All animals received pre-operative Clopidogrel (75 mg) and aspirin (81 mg) for 3 days prior to the procedure. On the day of the procedure, buprenorphine (0.008–0.011 mg/kg) was administered for analgesia, and propofol (4.0–5.0 mg/kg) and Isoflurane gas (2–3%) was administered for induction. Additionally, pre-operative prophylactic antibiotics (28–29 mg/kg Cefazolin) were given prior to the surgical cut down and regularly until the incision was closed. The index and terminal procedure were performed under inhalation anesthesia with isoflurane (0.5–2.5%) with continuous intraoperative monitoring. Analgesia was maintained throughout the procedure with Buprenorphine (0.3–0.7 mcg/kg/h). The coagulation status was monitored throughout the procedures by monitoring activated clotting time (ACT). Baseline ACT was measured, followed by heparin administration as appropriate to elevate the ACT ≥300 s with an effort to target an ACT between 300 and 500 s.

**Figure 1:**
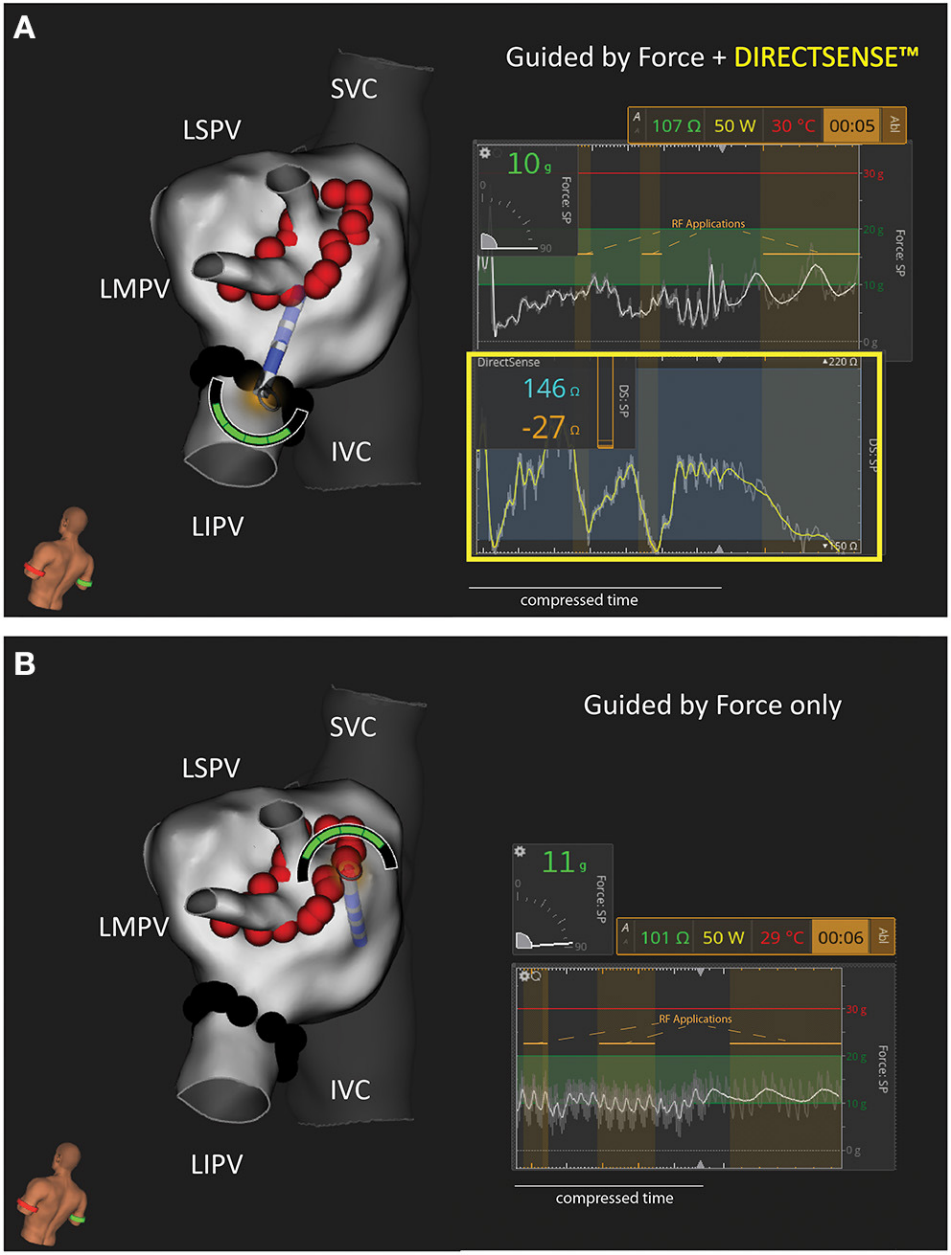
Representative Example of Study Workflows in the Canine Left Atrium. Two test conditions were used to isolate the left sided pulmonary veins in this study: **(A)** Force and DIRECTSENSE™ feedback visible during RF energy titration or **(B)** Only Force feedback visible during RF energy titration. In this example the LIPV was randomly assigned to **(A)** while the LSPV/LMPV was randomly assigned to **(B)**. *N* = 4 animals were treated with 50 W (as shown in this example). *N* = 4 separate animals were treated with 30 W. The force and local impedance traces include multiple sequential RF applications as highlighted by the gold overlays. IVC, inferior vena cava; LIPV, left inferior pulmonary vein; LMPV, left middle pulmonary vein; LSPV, left superior pulmonary vein; SVC, superior vein cava.

All study animals underwent an index procedure that included right atrial and left atrial (LA) electroanatomical maps using the IntellaMap Orion and Rhythmia HDx Mapping System (Boston Scientific, Marlbourough, MA). Venograms and intracardiac echocardiography (Vivid IQ, GE Healthcare) images of the target veins were acquired prior to ablation. Echocardiography was used to confirm target PVs were patent with a diameter of ≥9 mm. Isolation of the left superior PV and left inferior PV was performed using the Maestro 4000 and MetriQ Irrigation pump (Boston Scientific). In some cases, the superior PV branched into a superior and middle vein within the ostium. When this occurred, a larger isolation line was performed to encompass both the left superior and the left middle PV. All ablations were performed with the IntellaNav StablePoint Ablation Catheter (Boston Scientific). An 8.5F steerable sheath (Agilis, Abbott or Zurpaz, Boston Scientific) was used during all procedures. A point-by-point workflow was followed under the guidance of a tag distance tool with the Rhythmia HDx Mapping System to control inter-lesion distance (target ≤ 5 mm). Animals were randomized to a 30 W or 50 W RF power cohort. Within an animal, the target veins were randomized for order and feedback condition (i.e., CF alone/blind to LI or CF and LI visible). RF durations were determined at the discretion of the operator. Per protocol, the generator was set to a maximum of 20 s for 50 W or 30 s for 30 W. In the LI blinded condition, operators used their standard of care to titrate RF duration (i.e., terminating the generator earlier than the maximum duration) using electrogram attenuation, generator impedance and their experience in this model to determine when to terminate RF. When LI was visible, operators were given the guidance that (1) LI drops <20 Ω may be indicative of insufficient depth and (2) the probability of a steam pop increased with a LI drop of ≥65 Ω based on previous animal and explanted tissue studies. Given this guidance, both operators targeted 20–35 Ω as ideal for this model. In both conditions, the operators were free to adjust RF duration based on the CF feedback (magnitude and angle). Additionally, intracardiac echo was available to guide catheter manipulation. Two additional focal applications were applied to the LA wall in discrete locations away from the isolation lines. These applications were used only for histological assessment. Figure 1 includes a representative image of the user interface under each test condition.

Acute conduction block of the treated PVs was confirmed using entrance and/or exit block after a 20-min waiting period. If gaps were found, one round of touch-up applications was permitted, followed by a repeated 20 min waiting period. After verification of acute conduction block, animals were recovered under veterinary supervision and survived for 30 ± 5 days, during which the animals were closely monitored for adverse events.

At the terminal procedure, venograms were repeated to assess for potential PV stenosis of the treated veins. Electroanatomical maps were acquired and entrance and/or exit block of the PV ablation targets was evaluated prior to euthanasia. A full necropsy was performed on all subject animals and select tissue samples were processed and sent for histological assessment (StageBio Pathology).

All RF parameters including LI, CF, and RF duration were retrospectively exported from the mapping system for analysis. The starting LI and LI drop are calculated after the raw values are passed through a median filter with a window length of 1.5 s. Any application during which the sheath was detected to cover the distal ring electrode was excluded due to interaction with the CF and LI parameters. Continuous variables were reported as median with interquartile range (Q1–Q3). Linear correlation coefficients and statistical assessments were performed using Matlab 2019a (Mathworks). Multi-comparison tests were performed with a non-parametric one-way analysis of variance to determine statistical significance. Reported *p*-values measure the significance of the chi-square statistic, where *p* ≤ 0.05 is considered significant.

## Results

A total of *N* = 324 individual RF applications were delivered in the PV region across eight animals. All treated PVs were acutely isolated at the end of the index procedure and isolation was confirmed at the terminal procedure (16/16 veins). Representative examples of the electroanatomical LA maps pre- and post-ablation from the index and terminal procedure are included in the Supplementary Material. Only one isolation (superior/middle, 30 W: CF only) had a gap after the first waiting period and required additional RF applications. No incidents of PV stenosis (defined as ≥50% reduction in diameter on venograms) were noted in this study at the terminal procedure. Only one steam pop was observed in this study on intracardiac echocardiography during RF application with 50 W in the blinded to LI cohort after 6 s of ablation.

**Figure 2:**
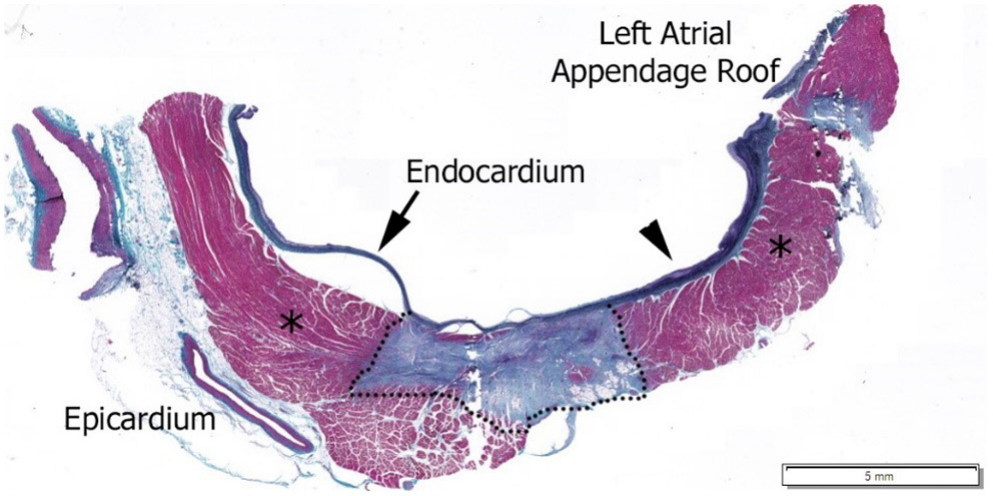
Hematoxylin and Eosin stained histological image of a 30 W, 9 s RF application with a 22 Ω drop in local impedance on the left atrium posterior wall. Dashed line outlines mature lesion, *Regions are normal myocardium as identified by pathologist.

Prior to RF application, LI was determined in the blood pool. For each individual RF application, the starting LI and average CF at RF start and the LI drop during RF delivery were acquired. Table 1 summarizes the median and interquartile range for CF and LI by experimental cohort. A similar CF was achieved in all workflows (median of 10.6–13.7 g). The median LI drop during RF ranged from 23 to 34 Ω depending on the ablation strategy. To understand the context of the observed LI drops, a tissue sample from a focal application in the LA is included in Figure 2. This cross-section, stained with hematoxylin and eosin, is excised from a lesion created with 30 W of RF energy for 9 s. The observed LI drop was 22 Ω. For a drop of this magnitude, only the thinner tissue is transmural in this cross section.

**Table 1:** Summary statistics of CF and LI for all RF applications (median and quartiles).

**Cohort**		**Starting contact force (g)**	**Pre-RF baseline local impedance (Ω)**	**Drop in local impedance (Ω)**
30 W	CF only	11.1 (8.0–15.0)	174.0 (152.0–193.5)	24.0 (18.0–35.0)
	CF + LI	10.6 (6.8–13.8)	170.0 (152.0–187.0)	23.0 (17.0–28.0)
50 W	CF only	12.6 (9.6–17.9)	165.0 (149.0–181.8)	30.0 (23.0–37.0)
	CF + LI	13.7 (9.7–17.7)	186.0 (171.0–218.0)	34.0 (30.0–43.0)

**Figure 3:**
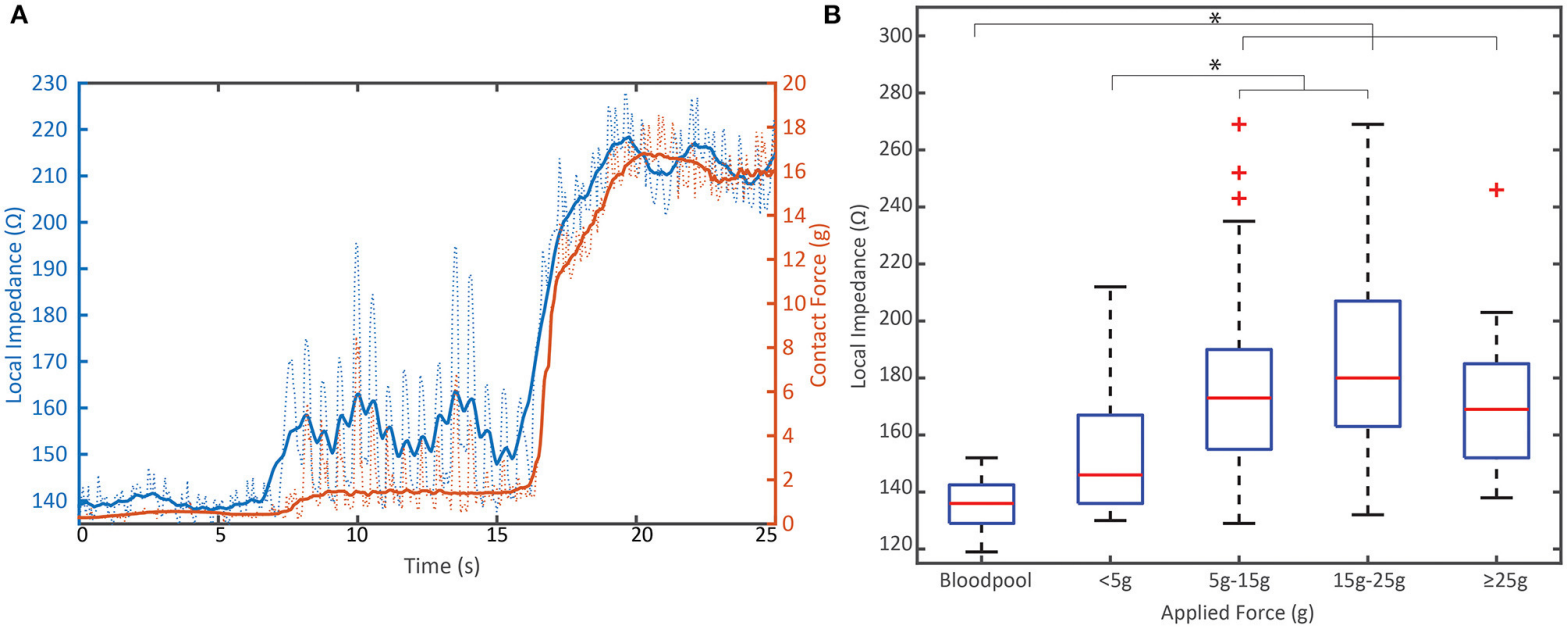
Relationship between mechanical load and electrical load prior to RF. **(A)** Representative example of the dynamic change in local impedance with increasing mechanical coupling between the tissue and tip electrode. Solid lines represent average force or local impedance; dashed lines represent raw traces measured at 20 Hz. **(B)** Distribution of the starting local impedance of all PVI RF applications by applied contact force compared to the local impedance in blood pool from *N* = 8 animals. Local impedance against tissue when >5 g is applied is significantly different than blood pool measurements (*p* < 0.001). Data displayed in box and whisker plot where box represents 25th to 75th percentile, Red line is the median, dashed lines represent the minimum and maximum and the red crosses are outliers defined as a value that is more than 1.5 times the interquartile range away from the bottom or top of the box. ^*^ indicates statistical significance.

CF and LI each measure a complimentary attribute of the coupling between the tip electrode and tissue. This relationship is illustrated in Figure 3. The time series demonstrates how both parameters increase as contact is increased in a single location. A video that highlights the dynamics of this interaction is also included in the Supplementary Material. Across the full dataset, the LI value in blood pool was statistically less than the coupling achieved when force exceeded 5 g (*p* < 0.001). LI values with contact ≥5 g were not statistically different from blood pool.

**Figure 4:**
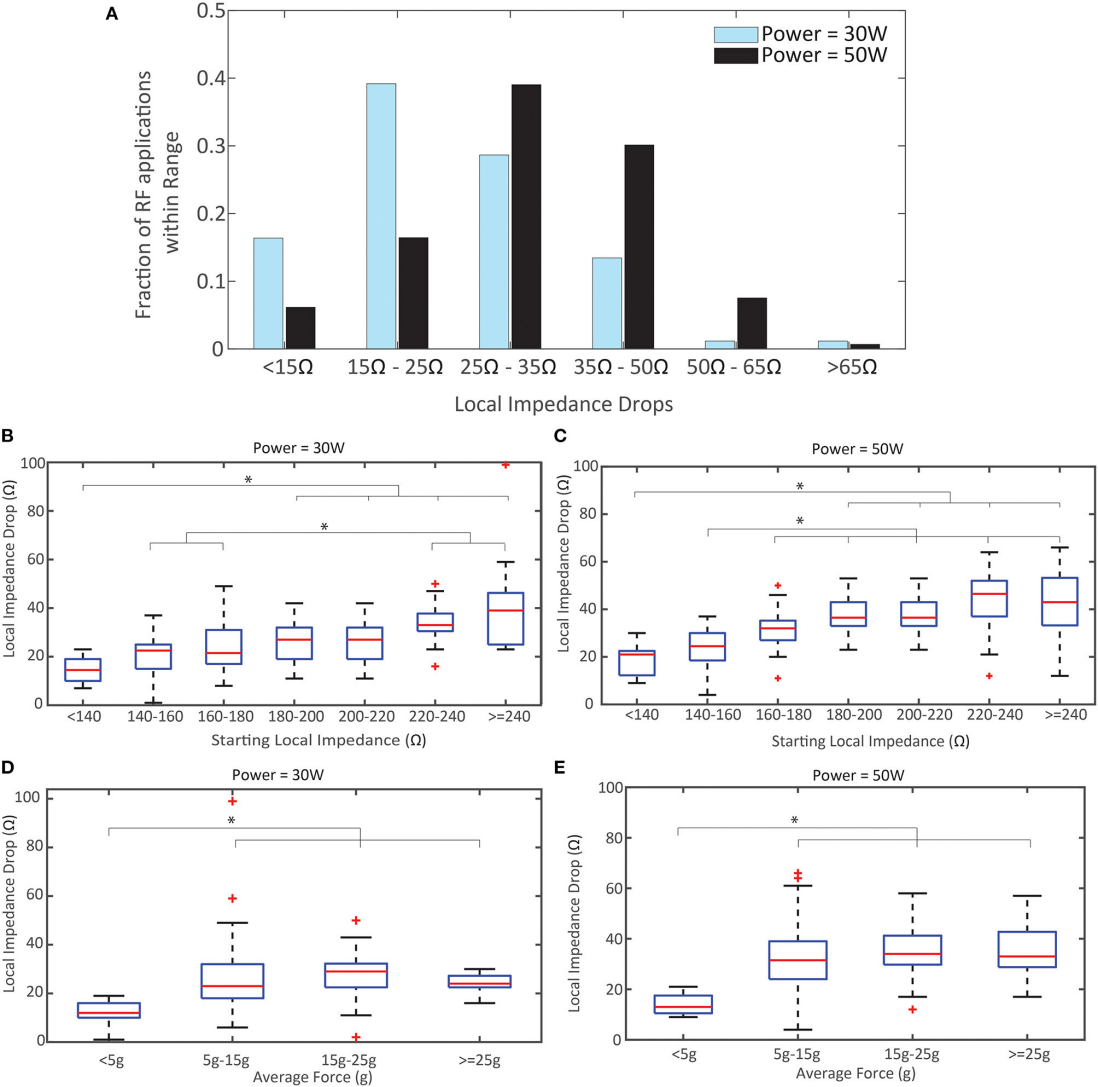
Local Impedance Drop by Power, Starting Local Impedance, and Force. **(A)** Distribution of Local Impedance Drops in the 50 W cohort compared to the 30 W. **(B,C)** Local Impedance Drop distribution based on the starting local impedance for the 30 W cohort **(B)** and 50 W **(C)**. Both demonstrate a direct relationship. **(D,E)** Local Impedance Drop distribution based the contact force for the 30 W cohort **(D)** and 50 W **(E)**. Both demonstrate a plateau in effect once force ≥5 g applied force. *p* < 0.001 for **(B-D)**; *p* = 0.0059 for **(E)**. ^*^ indicates statistical significance.

The distributions of LI drop during RF in each power cohort are displayed in Figure 4A. There is a demonstrated shift to larger drops when using 50 W compared to 30 W in this study. The proportion of drops that are <25 Ω decreased by >50% in the 50 W power cohort. This data was further segmented by whether the LI was visible to the user. When 30 W was used, 30/88 (34.1%) RF applications resulted in a LI drop <20 Ω if the user was blind to LI and 26/89 (29.2%) RF applications resulted in a LI drop <20 Ω if the user visualized LI. Conversely, when 50 W was used, 15/85 (17.6%) RF applications resulted in a LI drop <20 Ω if the user was blinded to LI compared to 2/62 (3.2%) if the user visualized LI.

In Figures 4B,C the relationship between the magnitude of the LI drop and the starting impedance is summarized for each power cohort (pooling LI visualization cohorts). The linear correlation coefficient is 0.59 for 30 W and 0.61 for 50 W, demonstrating a similar correlation between starting LI and observed LI drop regardless of power strategy. The LI drops with the lower starting impedance conditions are statistically different from the LI drops with the largest starting impedance for both power cohorts (*p* < 0.001 for 30 W, *p* < 0.001 50 W). Notably, the median drop with a starting LI <140 Ω for the 30 W cohort is less than the guidance of 20 Ω (14.5 Ω, R: 7–23 Ω) while the median drop in the same group for 50 W is >20 Ω (21 Ω, R: 9–30 Ω). The strong correlation with starting LI is contrasted by the relationship with CF in Figures 4D,E, where the linear correlation coefficient is 0.20 for 30 W and 0.27 for 50 W. In both cohorts, there is no statistical difference between the LI drop and CF once a minimum force of ≥5 g is achieved. Also, in both cohorts, the median drop observed with CF <5 g was less than the guidance of 20 Ω (12 Ω for 30 W and 13 Ω for 50 W).

**Figure 5:**
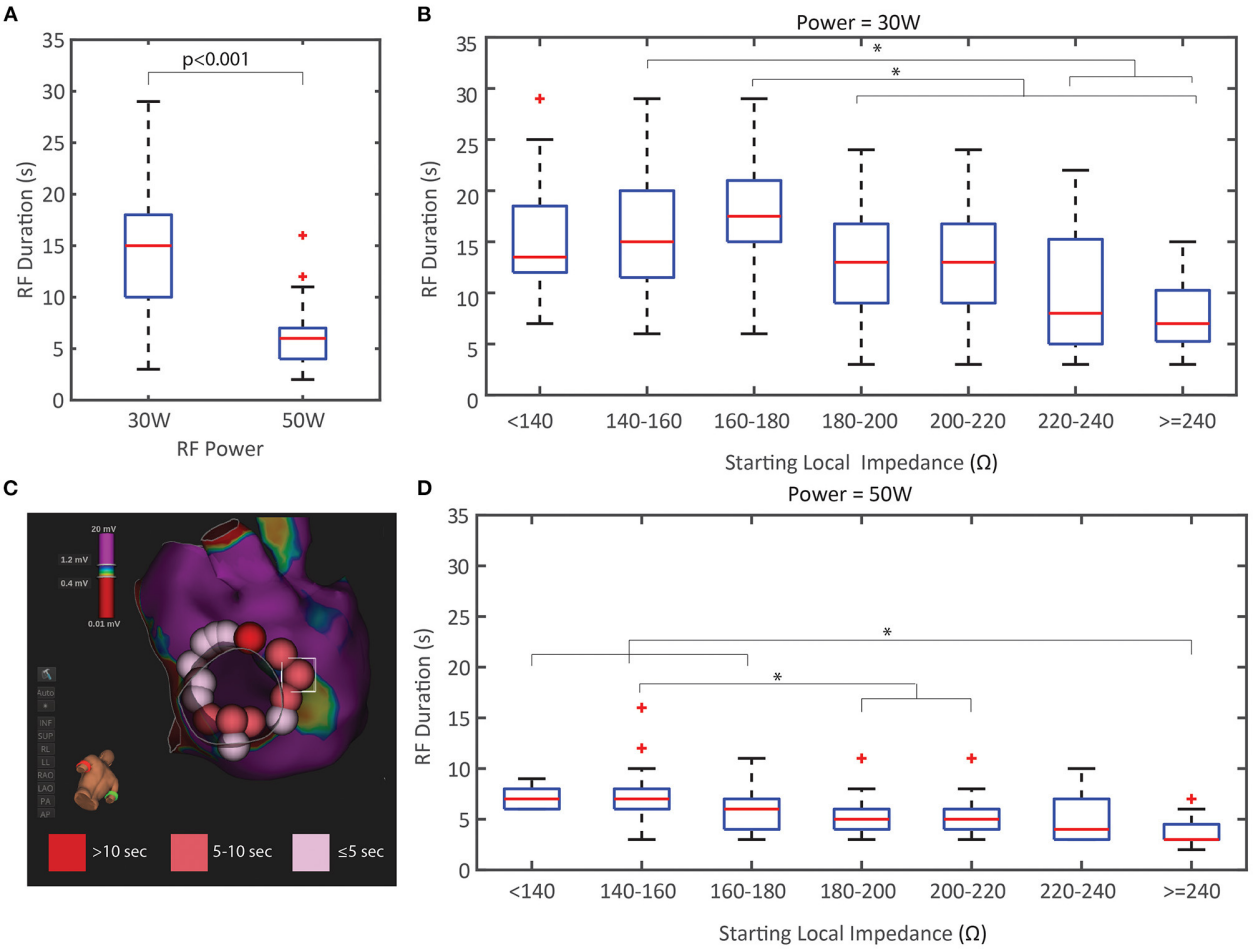
Duration of RF Energy Titration by Power and Starting Local Impedance. **(A)** RF duration was significantly less in the 50 W cohort compared to the 30 W. **(B,D)** RF duration distribution based on the starting local impedance for the 30 W cohort **(B)** and 50 W **(D)** both demonstrate an inverse relationship where higher starting impedances results in shorter RF durations. **(C)** Representative example of LIPV isolation at 50 W with ablation tags colored by RF time showing the spatial distribution of <5 s applications. ^*^ indicates statistical significance.

In addition to the relationship between the LI drop per power cohort, the effect on RF duration was explored in this study. Figure 5 compares the distribution of RF durations and the relationship with the starting LI per power cohort (including application with and without LI visible). As expected, the 50 W applications were significantly shorter than the 30 W applications (*p* < 0.001) and less overall RF energy was delivered to the tissue when an elevated power strategy was used. There is an inverse relationship between starting LI and RF duration in both cohorts where the shortest RF applications were observed when the starting LI was greatest. Additionally, a representative image of a left inferior PV isolation with the ablation tags colored by RF time highlights that a consecutive string of applications <5 s was sufficient for durable block in this model when using an elevated power strategy.

The dataset was further segmented by CF only or CF + LI to assess the effect of visualizing LI on the RF duration. Due to differences in anatomy, including vein size and variability in the middle PV branch location, only the inferior PV applications were used to assess RF duration per test condition. The inferior PV provided a more uniform target across the full cohort of animals. The results are summarized in Table 2. In both power cohorts, the median RF duration and the estimated RF energy decreased if LI was visible to the operator during RF application.

**Table 2:** RF duration and estimated energy for all LIPV RF applications (median and quartiles).

**Cohort**		**RF duration (s)**	**Estimated RF energy (J)**	***p*-value**
30 W	CF only	17.0 (15.3–19.3)	510.0 (457.5–577.5)	*p* = 0.009
	CF + LI	14.0 (9.8–16.8)	420.0 (292.5–487.5)	
50 W	CF only	6.0 (5.0–7.0)	300.0 (250.0–350.0)	*p* = 0.019
	CF + LI	4.0 (3.0–6.8)	200.0 (150.0–337.5)	

**Figure 6:**
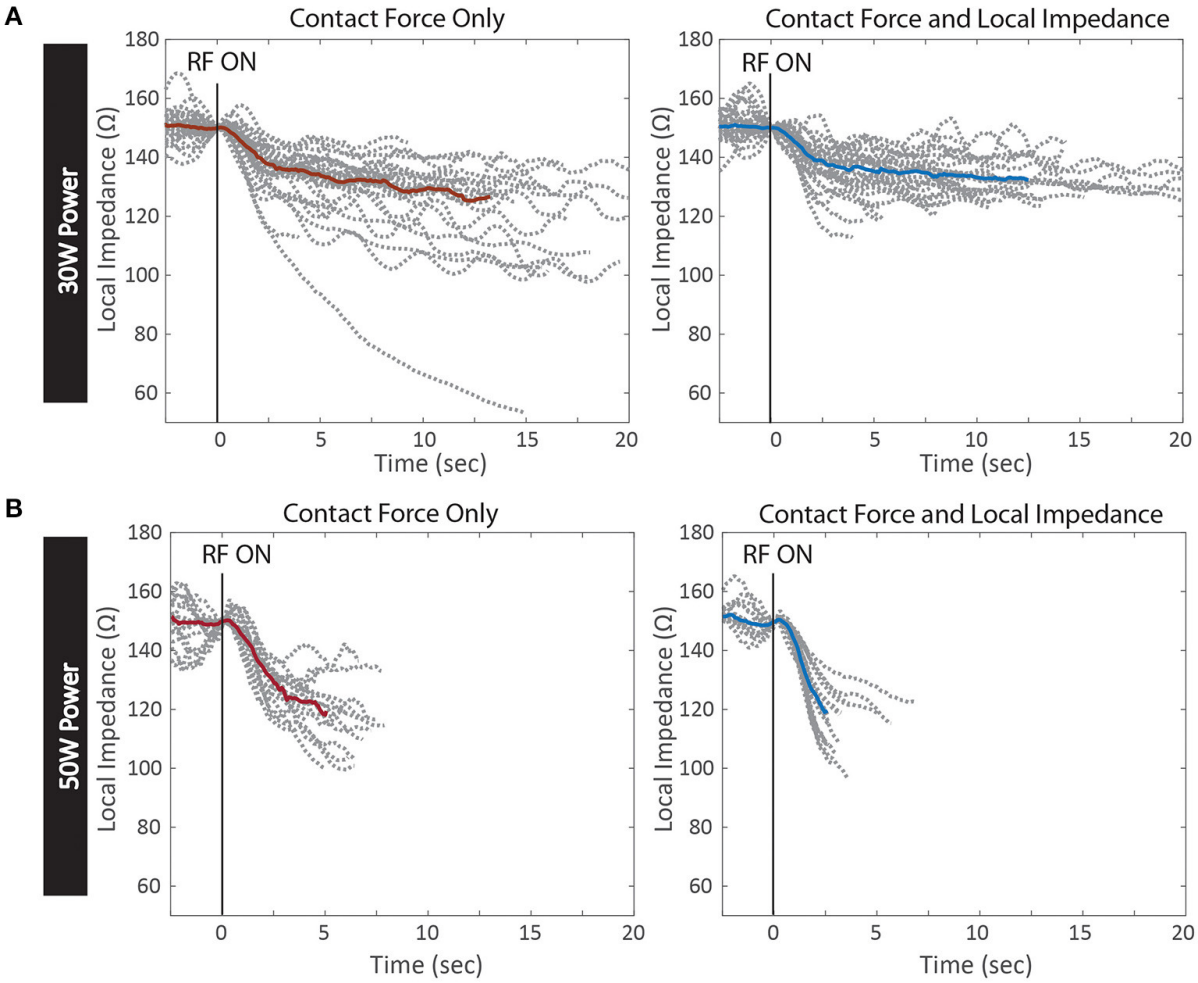
Kinetics of Local Impedance Drop per Test Conditions. Local impedance drops from RF applications of a representative PV isolation in each test condition. All drops are normalized to the same starting impedance at the onset of RF energy. The median drop in each condition is overlaid in red for Contact Force only and blue for Contact Force and Local Impedance **(A)** 30 W power cohort with Contact Force only (left) and Contact Force and Local Impedance (right) **(B)** 50 W cohort with Contact Force only (left) and Contact Force and Local Impedance (right).

The dynamic traces of the LI drop during RF ablation provide additional insights into the differences between the two power cohorts, beyond the magnitude of the drops alone. In Figure 6, the LI traces during all RF applications from the inferior PV isolation are displayed for a representative example from each cohort. All LI traces are normalized to the same arbitrary starting LI to compare the dynamics of the drop during RF delivery. LI at the time of RF onset was subtracted from all timepoints of the trace and then the traces were realigned to start at 150 Ω. The median drop is overlaid on top of the full population of LI traces. Greater variation in the time course of the drop during RF delivery is evident if the 30 W set of LI drops. Additionally, the maximum drop in these 4 examples occurs in the 30 W: CF only cohort, demonstrating that without feedback on the dynamics of tissue heating, overheating is possible, even with a nominal power strategy. It is evident that the population of traces that qualitatively look the most uniform comes from the 50 W: CF + LI cohort. With the additional feedback and the increased power, heating is more consistent, regardless of variability in the inherent physiological properties of the tissue.

## Discussion

This study demonstrated that while durable isolation can be achieved regardless of power strategy and available feedback, an elevated power strategy within the context of both CF and LI led to the most efficient titration of RF energy. Both the average RF duration per application and the overall delivered energy were statistically decreased when LI was visible and 50 W was used compared to a 50 W strategy within the context of force alone. Additionally, this study produced evidence that an elevated power strategy can overcome physiological variation in the resistive load due to normal heterogeneity in the target tissue. When the resistive load is high, the heating and corresponding LI drop is fast, and visualization of the dynamic drop provides a theoretical safety benefit. In this study access to the LI drop during RF led to the shortest RF durations when 50 W was used and the starting LI was >200 Ω. When LI was visible to the user, all drops >40 Ω were under 5 s in duration, regardless of power strategy. Moreover, the largest outlier LI drops were observed when the operator was blinded to the LI feedback. An example of a 99 Ω drop is included in Figure 6A for the 30 W cohort, which is much greater than the guidance for the optimal range for this catheter (65 Ω).

Conversely, when the resistive load is low, the heating and corresponding drop is slow and visualization of the drop provides insight that allows for more controlled titration of energy. When the starting LI is <180 Ω, RF durations were nearly twice the duration where the starting LI was closer to 240 Ω. When 30 W was used, it was difficult to achieve an LI drop near the proposed guidance of 20 Ω when the starting resistive load was low. This suggests that 50 W may be able to overcome some of the challenges of decreased tissue resistivity that 30 W cannot, even if delivered for a longer duration or as a larger total energy dose. With 50 W and the additional LI feedback (both the initial value and the dynamic changes during energy delivery), the LI drops are visually more consistent and reproducible, as the feedback reflects the biophysics of the tissue. This feedback provides an opportunity to customize the strategy based on the tissue rather than using a uniform approach throughout that disregards with normal tissue variation. Whereas, ablating with 30 W introduced more variability in the tissue response (i.e., variability in the slope of heating, drops achieved, etc.) compared to ablating with an elevated power strategy.

The LI metric used in this study is enabled through the creation of an electric field using only electrodes that are on the ablation catheter. This field is sensitive to the dynamic dielectric properties of the cardiac tissue, including changes that occur during tissue heating, based on many of the same principles behind the long-demonstrated utility of systemic generator impedance (Wang et al., 1995; Avitall et al., 1997). However, when using generator impedance, extracardiac components including bulk resistivity within the thorax can add to, mask, or attenuate the dynamic range of the local interface impedance between the electrode tip and the myocardium. Any changes to respiration or the fluid status throughout a case and shifts in the measurement frequency, may all negatively impact the accuracy of the generator impedance feedback as an indicator of the electrical conductivity surrounding the electrode.

LI, as enabled by the DIRECTSENSE technology in the Rhythmia HDx Mapping System at 14.5 kHz, reduces bulk elements from the impedance circuit and enables the direct measurement of the local electrode-tissue impedance changes. The greater dynamic range also allows for more practical interpretation of differences in the magnitude and the kinetics of the LI drop during RF. It is important to note that the local impedance measure reflects both the impedance of the tissue and the nearby blood, depending on the coupling between the target tissue and the tip electrode. The LI measure is independent of the control system for the generator and therefore does not modulate the current output. The advantage over generator impedance is evident in the results of this study when comparing the RF applications with both CF and LI to CF alone, where generator impedance was also available to the operator. Additional studies are required to confirm that the potential advantage of using LI with an elevated power strategy to further reduce RF durations translates to the clinical environment. However, the data from this study demonstrate that there may be potential for clinical efficiency gain with regard to shorter RF and procedure times when these two complimentary variables are used in tandem. In addition, it gives some clinical insight with regard to ideal titration of power and ablation duration in tissues with lower resistivity load (i.e., lower local impedance), even when starting contact force may be constant.

This study also investigated the effect of CF on LI drops. Regardless of the power strategy employed, the results demonstrate that if a minimum force of 5 g is not achieved it is difficult to achieve sufficient heating. However, once the force exceeds 5 g there is no correlation between CF and tissue heating as measured by the LI drop. This is consistent with recent findings on the role of CF minimizing occurrences of insufficient contact but not providing more dynamic value during delivery to guide energy titration (Makimoto et al., 2014; Calkins et al., 2018). Similarly, a minimum force led to reduced generator impedance drops compared to increased force levels in a study of 30 patients, where a force <10 grams was the smallest force threshold analyzed (Williams et al., 2015). Once a minimum force is achieved, increased force only does not heavily influence the tissue response to RF energy, whereas increased resistive load does. This highlights the complimentary nature of the CF and LI together to provide a more robust understanding of the tissue-tip electrode interface.

## Limitations

Tissue in this model is thinner than most clinical conditions. All RF durations should be interpreted within the context of thinner tissue, which requires shorter duration RF applications on average compared to the clinical setting. Additionally, although there is heterogeneity in the resistive properties of the tissue in this study, the myocardial tissue in these animals is young and healthy. The variation in resistive load and the variation in the dynamics of heating may be increased in diseased tissue (i.e., infiltration of fibrosis or fat, existing lesions from previous ablations, co-morbidities that affect the tissue composition, etc.).

## Conclusion

Local impedance and contact force are complimentary variables that each provide information that the other cannot. CF appears to be most useful to assess conditions that are favorable to beginning an ablation lesion (i.e., mechanical interaction with tissue and electrode stability). LI offers additional insight about the resistive load present in this starting condition, allowing the user to select the most appropriate power while providing some *a priori* prediction of expected ablation time. In addition, it provides invaluable real-time feedback to changes in the condition of the local tissue, which can help the user determine when to terminate RF energy based on LI drops achieved at the selected power.

## Data Availability Statement

The datasets presented in this article are not readily available because this data was also submitted for regulatory approval. Requests for access will be handled on a per case basis. Requests to access the datasets should be directed to Jason Hamann, jason.hamann@bsci.com.

## Ethics Statement

The animal study was reviewed and approved by Institutional Animal Care and Use board of Boston Scientific Corp.

## Author Contributions

SG, JPM, and JDM performed and verified the analysis and data interpretation. JPM and JDM performed all ablations, determined energy titration strategies, and assessed all determinations of block. AS, MA-C, and VK served as animal model experts, assessed animals during recovery, and oversaw independent audit of data. All listed authors contributed to the design of the study protocol, data acquisition, and review of manuscript.

## Funding

This work was supported by Boston Scientific Corporation.

## Conflict of Interest

SG, AS, VK, MA-C, LL, and KM are salaried employees of Boston Scientific. The remaining authors declare that the research was conducted in the absence of any commercial or financial relationships that could be construed as a potential conflict of interest.

## Publisher's Note

All claims expressed in this article are solely those of the authors and do not necessarily represent those of their affiliated organizations, or those of the publisher, the editors and the reviewers. Any product that may be evaluated in this article, or claim that may be made by its manufacturer, is not guaranteed or endorsed by the publisher.

## References

[B1] Avitall B. Mughal K. Hare J. Helms R. Krum D. (1997). The effects of electrode-tissue contact on radiofrequency lesion generation. Pacing Clin. Electrophysiol. 20, 2899–2910. 10.1111/j.1540-8159.1997.tb05458.x 9455749

[B2] Barkagan M. Contreras-Valdes F. M. Leshem E. Buxton A. E. Nakagawa H. Anter E. (2018). High-power and short-duration ablation for pulmonary vein isolation: safety, efficacy, and long-term durability. J. Cardiovasc. Electrophysiol. 29, 1287–1296. 10.1111/jce.13651 29846987

[B3] Calkins H. Hindricks G. Cappato R. Kim Y.-H. Saad E. B. Aguinaga L.. (2018). 2017 HRS/EHRA/ECAS/APHRS/SOLAECE expert consensus statement on catheter and surgical ablation of atrial fibrillation. Ep Europace 20, e1–e160. 10.1093/europace/eux274 29016840 PMC5834122

[B4] Das M. Luik A. Shepherd E. Sulkin M. Laughner J. Oesterlein T.. (2021). Local catheter impedance drop during pulmonary vein isolation predicts acute conduction block in patients with paroxysmal atrial fibrillation: initial results of the LOCALIZE clinical trial. EP Europace. 23, 1042–1051. 10.1093/europace/euab004 33550380 PMC8286855

[B5] Garrott K. Laughner J. Gutbrod S. Sugrue A. Shuros A. Sulkin M.. (2020). Combined local impedance and contact force for radiofrequency ablation assessment. Heart Rhythm 17, 1371–1380. 10.1016/j.hrthm.2020.03.016 32240822

[B6] Gunawardene M. Münkler P. Eickholt C. Akbulak R. Ö. Jularic M. Klatt N.. (2019). A novel assessment of local impedance during catheter ablation: initial experience in humans comparing local and generator measurements. Europace 21(Suppl. 1): i34–i42. 10.1093/europace/euy273 30801126

[B7] Haines D. E. (1991). Determinants of lesion size during radiofrequency catheter ablation: The role of electrode-tissue contact pressure and duration of energy delivery. J. Cardiovasc. Electrophysiol. 2, 509–515. 10.1111/j.1540-8167.1991.tb01353.x

[B8] Kimura M. Sasaki S. Owada S. Horiuchi D. Sasaki K. Itoh T.. (2014). Comparison of lesion formation between contact force-guided and non-guided circumferential pulmonary vein isolation: a prospective, randomized study. Heart Rhythm 11, 984–991. 10.1016/j.hrthm.2014.03.019 24657428

[B9] Makimoto H. Lin T. Rillig A. Metzner A. Wohlmuth P. Arya A.. (2014). *In vivo* contact force analysis and correlation with tissue impedance during left atrial mapping and catheter ablation of atrial fibrillation. Circ. Arrhyth. Electrophysiol. 7, 46–54. 10.1161/CIRCEP.113.000556 24363353

[B10] Marijon E. Fazaa S. Narayanan K. Guy-Moyat B. Bouzeman A. Providencia R.. (2014). Real-time contact force sensing for pulmonary vein isolation in the setting of paroxysmal atrial fibrillation: procedural and 1-year results. J. Cardiovasc. Electrophysiol. 25, 130–137. 10.1111/jce.12303 24433324

[B11] Martinek M. Lemes C. Sigmund E. Derndorfer M. Aichinger J. Winter S.. (2012). Clinical impact of an open-irrigated radiofrequency catheter with direct force measurement on atrial fibrillation ablation. Pacing Clin. Electrophysiol. 35, 1312–1318. 10.1111/j.1540-8159.2012.03503.x 22946636

[B12] Natale A. Reddy V. Y. Monir G. Wilber D. J. Lindsay B. D. McElderry H. T.. (2014). Paroxysmal AF catheter ablation with a contact force sensing catheter: results of the prospective, multicenter SMART-AF trial. J. Am. Coll. Cardiol. 64, 647–656. 10.1016/j.jacc.2014.04.072 25125294

[B13] Neuzil P. Reddy V. Y. Kautzner J. Petru J. Wichterle D. Shah D.. (2013). Electrical reconnection after pulmonary vein isolation is contingent on contact force during initial treatment: results from the EFFICAS I study. Circ. Arrhyth. Electrophysiol. 6, 327–333. 10.1161/CIRCEP.113.000374 23515263

[B14] Sigmund E. Puererfellner H. Derndorfer M. Kollias G. Winter S. Aichinger J.. (2015). Optimizing radiofrequency ablation of paroxysmal and persistent atrial fibrillation by direct catheter force measurement—a case-matched comparison in 198 patients. Pacing Clin. Electrophysiol. 38, 201–208. 10.1111/pace.12549 25469738

[B15] Sulkin M. S. Laughner J. I. Hilbert S. Kapa S. Kosiuk J. Younan P.. (2018). Novel measure of local impedance predicts catheter–tissue contact and lesion formation. Circ. Arrhyth. Electrophysiol. 11:e005831. 10.1161/CIRCEP.117.005831 29618475

[B16] Wang D. Hulse J. Walsh E. Saul J. (1995). Factors influencing impedance during radiofrequency ablation in humans. Chin. Med. J. 108, 450–455. 7555256

[B17] Williams S. E. Harrison J. Chubb H. Bloch L. Ø. Andersen N. P. Dam H.. (2015). The effect of contact force in atrial radiofrequency ablation: electroanatomical, cardiovascular magnetic resonance, and histological assessment in a chronic porcine model. JACC Clin. Electrophysiol. 1, 421–431. 10.1016/j.jacep.2015.06.003 29759471

