# Supplementary material for: Improved Ablation Efficiency in PVI Guided by Contact Force and Local Impedance: Chronic Canine Model
Source: Front Physiol. 2022 Jan 10;12:808541. doi: 10.3389/fphys.2021.808541 (PMC8784686; doi:10.3389/fphys.2021.808541)
Supplement: Supplementary file 2 [file Data_Sheet_1.PDF]

**Supplemental Material**

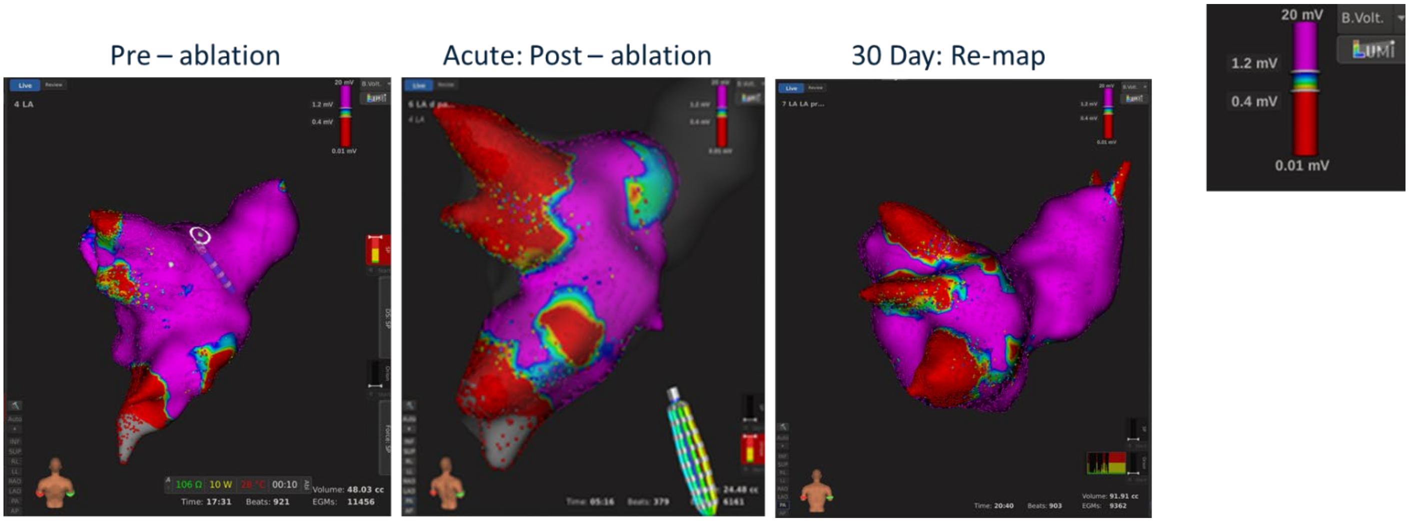


Representative Examples of Voltage Maps. Power = 30W; LSPV + LMPV Blinded to LI, LIPV with Force and LI

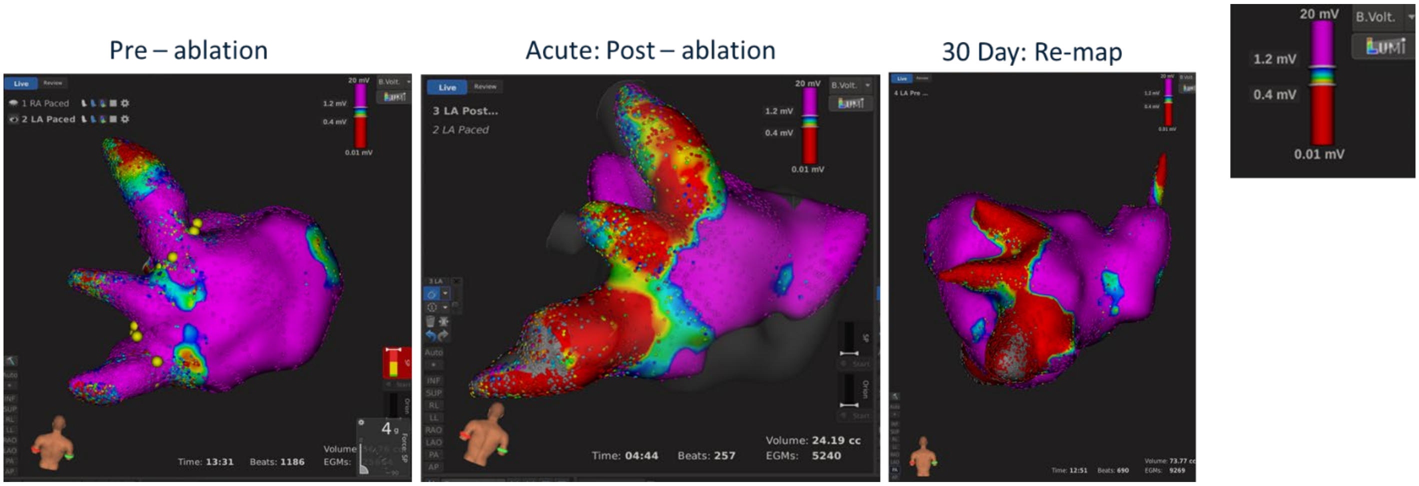


Representative Examples of Voltage Maps. Power = 50W; LIPV Blinded to LI, LSPV +LMPV with Force and LI
